# Wogonin Inhibits Apoptosis and Necroptosis Induced by Nephropathogenic Infectious Bronchitis Virus in Chicken Renal Tubular Epithelial Cells

**DOI:** 10.3390/ijms25158194

**Published:** 2024-07-27

**Authors:** Qiurong Qi, Ying Li, Mengbing Ding, Cheng Huang, Salma Mbarouk Omar, Yan Shi, Ping Liu, Gaofeng Cai, Zhanhong Zheng, Xiaoquan Guo, Xiaona Gao

**Affiliations:** 1 Jiangxi Provincial Key Laboratory for Animal Health, Institute of Animal Population Health, College of Animal Science and Technology, Jiangxi Agricultural University, Nanchang 330045, China; 2 School of Computer and Information Engineering, Jiangxi Agricultural University, Nanchang 330045, China

**Keywords:** wogonin, nephropathogenic infectious bronchitis virus, renal tubular epithelial cells, apoptosis, necroptosis

## Abstract

NIBV is an acute and highly contagious virus that has a major impact on the poultry industry. Wogonin, as a flavonoid drug, has antiviral effects, but there have been no reports indicating its role in renal injury caused by NIBV infection. The aim of this study is to investigate the antiviral effect of wogonin against NIBV. Renal tubular epithelial cells were isolated and cultured, and divided into four groups: Con, Con+Wog, NIBV and NIBV+Wog. We found that wogonin significantly inhibited the copy number of NIBV and significantly alleviated NIBV-induced cell apoptosis and necrosis. Moreover, wogonin inhibited the reduction in mitochondrial membrane potential and the aberrant opening of mPTP caused by NIBV. In conclusion, wogonin can protect renal tubular epithelial cells from damage by inhibiting the replication of NIBV and preventing mitochondrial apoptosis and necroptosis induced by NIBV.

## 1. Introduction

The infectious bronchitis virus (IBV) is the cause of infectious bronchitis (IB), an acute and highly contagious respiratory infectious disease [1]. The nephropathogenic infectious bronchitis virus (NIBV) stands as a commonly occurring strain of IBV among the commercial poultry industry. In recent years, the prevalence of NIBV has been on the rise in the commercial poultry industry [2], and NIBV has already caused significant economic losses to the poultry industry. NIBV, classified as a γ-coronavirus in the coronavirus family, mainly affects the respiratory, digestive, urinary, and reproductive systems [3]. The presenting symptoms are cough, sneezing, decreased laying rate and egg quality [4]. NIBV primarily targets the kidneys, causing swelling and paleness after infection. Although vaccines have been used in the poultry industry, there are some side effects. Hence, the pressing demand lies in elucidating the mechanisms underlying NIBV-induced kidney injury and creating efficacious therapeutic approaches.

Apoptosis and necrosis are involved in the process of body damage caused by various viruses. Studies have reported that viruses often lead to mitochondrial apoptosis and necroptosis. PRRSV (Porcine Reproductive and Respiratory Syndrome Virus) has been demonstrated to induce apoptosis of lung and immune organs and to compromise the host immune system [5]. SARS-CoV-2 can cause apoptosis in seminiferous tubules and necrosis in the testis [6]. Human coronavirus (HCoV) infection induces stress response, autophagy, apoptosis, and innate immunity [7]. Apoptosis, a non-inflammatory type of cell death, is crucial for maintaining homeostasis, facilitating tissue development, and regulating the immune system. It selectively eliminates cells that have become redundant or no longer serve a purpose [8]. Apoptosis is categorized into three pathways: mitochondrial, endoplasmic reticulum and death receptor pathways. These three pathways ultimately lead to the activation of Caspase-3 and the formation of apoptotic bodies [9]. Necroptosis represents a regulated modality of necrotic cell demise, mediated by receptor-interacting protein kinases (RIPKs) [10]. RIPK3 serves as a pivotal element at the heart of the necroptosome complex, encompassing both RIPK1 and MLKL, playing a crucial role in its function [11]. Programmed cell necrosis can be induced by various factors such as viruses and chemotherapy drugs. Studies have shown that both the influenza virus and severe acute respiratory syndrome coronavirus 2 (SARS-CoV-2) can induce organ apoptosis and programmed death in mice [12,13]. Therefore, it may be feasible to seek drugs that prevent and control NIBV by inhibiting apoptosis and necroptosis.

Wogonin (C_16_H_12_O_5_), a flavonoid and flavonoid compound featuring O-methylation, has been discovered in the plant known as scutellaria [14]. The flavonoid wogonin exhibits a variety of pharmacological effects, for example with antiviral, antioxidant, anticancer and anti-inflammatory properties [15,16]. Recent studies have demonstrated that wogonin can protect renal cells in diabetic nephropathy by focusing on bcl-2-mediated autophagy and apoptosis [17], and that it can also reduce cisplatin-induced renal injury in mice by targeting ripk1-mediated necroptosis [18]. Furthermore, wogonin has shown antiviral activity against porcine epidemic diarrhea virus (PEDV) [19], herpes simplex virus type 1 (HSV-1), herpes simplex virus type 2 (HSV-2) [20], and hepatitis B virus (HBV) [21]. However, the role of wogonin in mitochondrial apoptosis and necroptosis in renal tubular epithelial cells infected with NIBV has not yet been determined.

This study utilizes a model of NIBV-infected renal tubular epithelial cells to investigate the apoptotic and necrotic effects induced by NIBV infection in renal tubular epithelial cells, as well as the effects of wogonin.

## 2. Results

### 2.1. Wogonin Inhibits NIBV-Induced Cell Death

**Figure 1:**
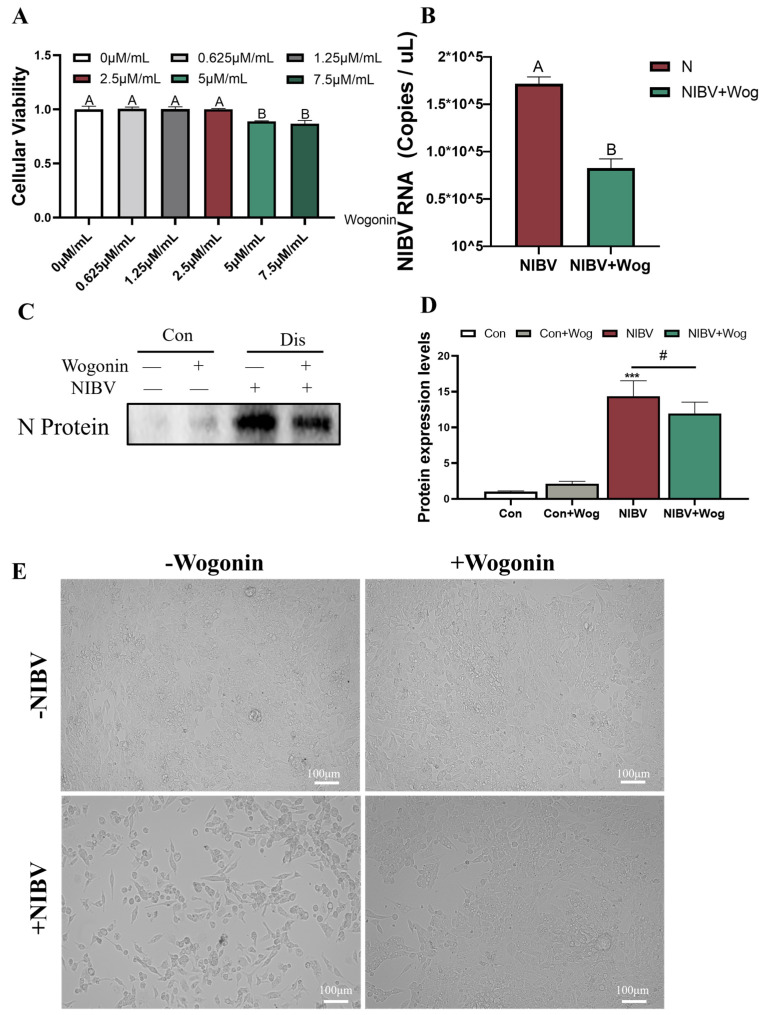
Effects of wogonin on NIBV−induced cell death. (**A**) The impact of different concentrations of wogonin on the activity of renal tubular epithelial cells was determined using the CCK8 method (*n* = 12). (**B**) The influence of wogonin on the copy number of NIBV in renal tubular epithelial cells (*n* ≥ 4). (**C,D**) The protein levels of N protein of four different groups (*n* = 3). (**E**) The morphology of NIBV-infected renal tubular epithelial cells after adding wogonin (Scale bar = 100 μm). (A,B) for significance level with *p* < 0.01. “*” indicates the comparison with the Con group (*** *p* < 0.001), “#” indicates the comparison with the NIBV group (# *p* < 0.05).

In order to detect the suitable concentration of wogonin, renal tubular epithelial cells were incubated with varying concentrations of wogonin (0, 0.625, 1.25, 2.5, 5, and 7.5 μM/mL). As shown in Figure 1A, a concentration of 2.5 μM/mL showed no significant impact on the cells. Therefore, we chose 2.5 μM/mL for subsequent experimental procedures. To determine whether wogonin has an anti-NIBV effect, we detected viral replication. According to Figure 1B, the virus copy number of the NIBV+Wog group was less than that of the NIBV group. The results of Western blotting indicated a significantly reduced expression level of N protein in the NIBV+Wog group compared to the NIBV group, suggesting that wogonin also inhibited virus replication at the protein level (Figure 1C,D). Also, it was observed that NIBV caused the death of renal tubular epithelial cells. However, this effect was significantly reduced by wogonin (Figure 1E). Therefore, wogonin has the potential to counteract the toxicity of NIBV and prevent cell death.

### 2.2. Wogonin Attenuates the Apoptosis and Necrosis of Renal Tubular Epithelial Cells Induced by NIBV

**Figure 2:**
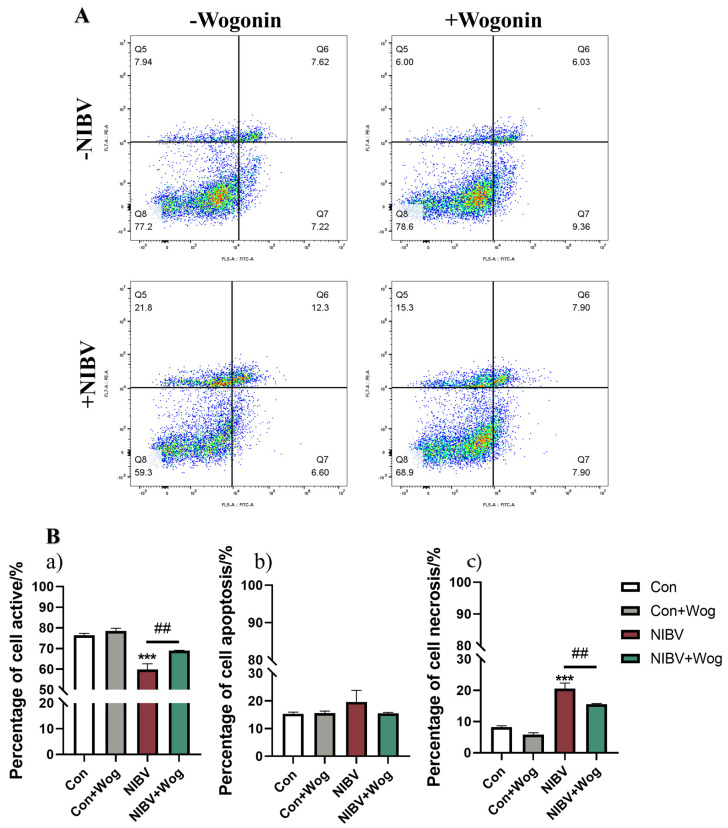
The effect of wogonin treatment on cell apoptosis and necrosis. (**A**) Flow cytometric analysis of primary renal tubular epithelial cells stained with PI and Annexin V (*n* = 3). (**B**) A quantitative map of cell viability: cell active (**a**), cell apoptosis (**b**) and cell necrosis (**c**). “*” indicates the comparison with the Con group (*** *p* < 0.001), “#” indicates the comparison with the NIBV group (## *p* < 0.01).

Viral infection often leads to cell death [22], with apoptosis being a significant form of such cellular demise [23]. Apoptosis and necrosis are widely involved in NIBV-induced cell death. Using flow cytometry, the effects of wogonin on cell apoptosis and necrosis induced by NIBV infection were examined. PI/Annexin V staining was performed on primary renal tubular epithelial cells. After NIBV infects the renal tubular epithelial cells, their vitality significantly decreases, and apoptosis and necrosis of the cells occur. Compared with the NIBV group, the proportion of apoptotic and necrotic cells in the NIBV+Wog group is reduced (Figure 2A,B). Therefore, wogonin administration alleviated the apoptosis and necrosis of renal tubular epithelial cells caused by NIBV.

### 2.3. Effects of Wogonin on Mitochondrial Membrane Potential

**Figure 3:**
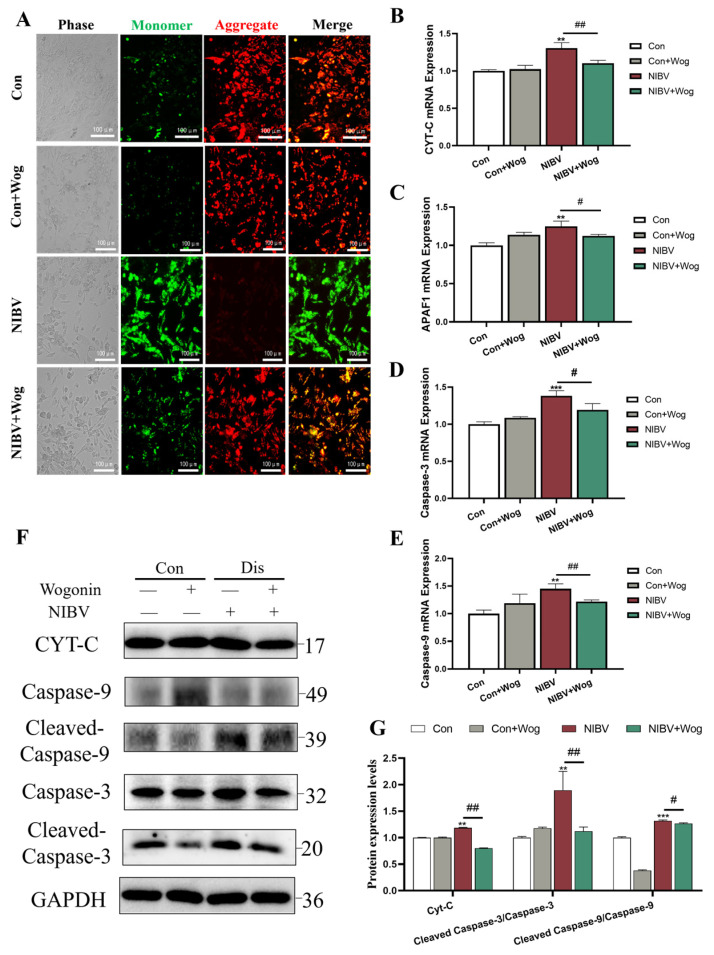
The alleviation effect of wogonin on NIBV-induced apoptosis. (**A**) Effects of wogonin on mitochondrial membrane potential decline after NIBV infection (Scale bar = 100 μm). (**B**–**E**) Mitochondrial apoptosis gene expression was measured in renal tubular epithelial cells after NIBV infection (*n* = 3). (**F**,**G**) Effect of wogonin on expression of mitochondrial apoptosis proteins (CYT-C, APAF1, Caspase-3 and Caspase-9) after viral infection (*n* = 3). “*” indicates the comparison with the Con group (** *p* < 0.01, *** *p* < 0.001), “#” indicates the comparison with the NIBV group (# *p* < 0.05, ## *p* < 0.01).

A reduction in mitochondrial membrane potential indicates early apoptosis. To investigate whether wogonin can reduce the apoptosis induced by NIBV, we first measured the membrane potential. In normal circumstances, the mitochondrial membrane potential remains elevated, resulting in JC-1 existing as polymers and emitting strong red fluorescence. However, during apoptosis, a drop in mitochondrial membrane potential prompts JC-1 to adopt a monomeric form, intensifying green fluorescence. As depicted in Figure 3A, the Con group exhibited strong red fluorescence that was gathered in the mitochondria and cytoplasm, while the NIBV group showed a transformation of JC-1 from an aggregate to a monomer, resulting in a significant weakening of red fluorescence and an enhancement of green fluorescence. This indicates the occurrence of apoptosis. After treatment with wogonin, the red fluorescence intensity increased while the green fluorescence intensity decreased. This suggests that wogonin may alleviate the decrease in mitochondrial membrane potential induced by NIBV.

### 2.4. Effects of Wogonin on the Expression Levels of Apoptosis-Related Genes and Proteins Induced by NIBV

To further elucidate the impact of wogonin on NIBV-induced apoptosis, we analyzed the expression changes of apoptosis-related genes and proteins. In Figure 3B,E, the NIBV group demonstrated significant increases in mRNA expression levels of APAF1 (apoptotic protease activating factor 1), CYT-C (cytochrome c), Caspase-3, and Caspase-9 compared to the Con group (*p* < 0.01 or *p* < 0.001). Moreover, wogonin treatment also significantly reduced the mRNA levels of genes related to apoptosis (APAF1, CYT-C, Caspase-3 and Caspase-9) (*p* < 0.05 or *p* < 0.01). Western blot assays also confirmed that wogonin significantly reduced the expression of apoptosis-related proteins, induced by NIBV in renal tubular epithelial cells (Figure 3F,G). The results of the aforementioned genes and proteins indicate that wogonin can alleviate the apoptosis of renal tubular epithelial cells induced by NIBV.

### 2.5. Wogonin Can Alleviate the Increase in mRNA and Protein Expression Levels of Necrosis-Associated Factors Induced by NIBV

**Figure 4:**
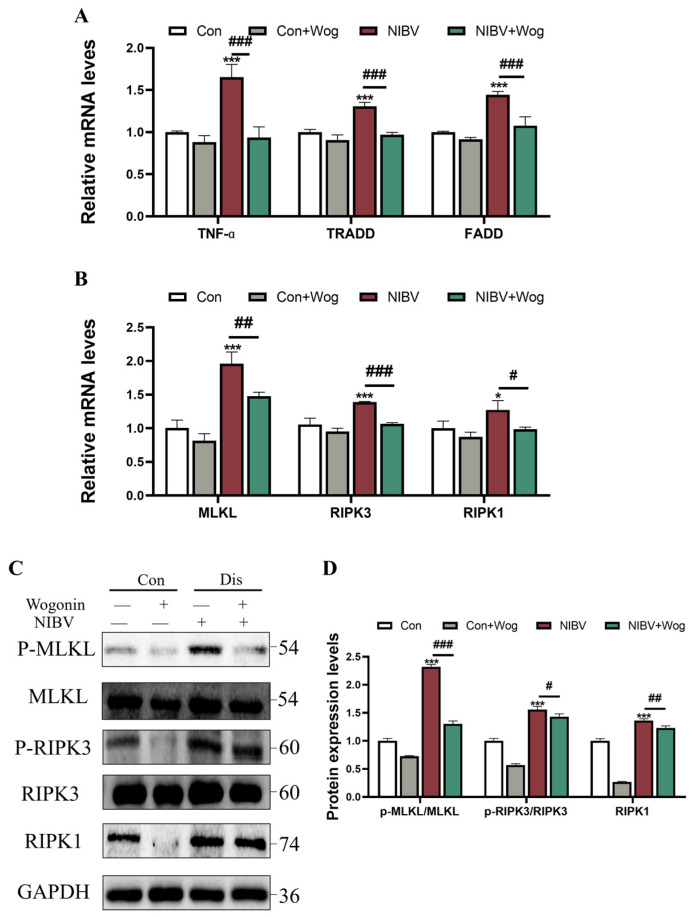
The alleviation effect of wogonin on NIBV−induced necroptosis. (**A**,**B**) Effects of wogonin on mRNA expression levels of necroptosis-related genes (TNF-α, TRADD, FADD, MLKL, RIPK1, RIPK3) induced by NIBV (*n* = 3). (**C**,**D**) The impact of wogonin on the expression of necroptosis-related proteins (MLKL, RIPK1, RIPK3) induced by NIBV (*n* = 3). “*” indicates the comparison with the Con group (* *p* < 0.05, *** *p* < 0.001), “#” indicates the comparison with the NIBV group (# *p* < 0.05, ## *p* < 0.01, ### *p* < 0.001).

To further detect the impact of wogonin on the necroptosis signaling pathway caused by NIBV, we assessed the mRNA expression levels of genes associated with necroptosis. The results in Figure 4A,B demonstrated that wogonin exerts a notable suppressive effect on the expression of TNF-α (Tumor necrosis factor alpha), TRADD (Tumor necrosis factor receptor-associated death domain protein), FADD (Fas-associated protein with death domain) and RIPK3 (Receptor interacting protein kinase 3) induced by NIBV (*p* < 0.001). Wogonin significantly downregulated RIPK1 (Receptor interacting protein kinase 1) and MLKL (Mixed lineage kinase domain like-protein) genes (*p* < 0.05 or *p* < 0.01). The results indicated that wogonin could inhibit the up-regulation of necroptosis-related genes caused by NIBV. Western blot analysis (Figure 4C,D) demonstrated that wogonin inhibits the increased expression of RIPK1 protein induced by NIBV infection (*p* < 0.01), along with the ratios of P-RIPK3/RIPK3 and P-MLKL/MLKL protein expression (*p* < 0.05 or *p* < 0.001). These results demonstrate that wogonin can reduce NIBV-induced programmed cell necroptosis.

### 2.6. Wogonin Inhibits RIPK3 Expression

**Figure 5:**
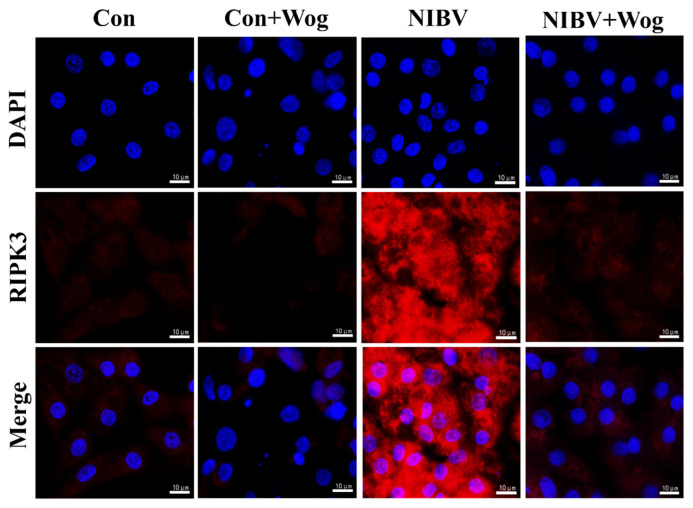
The expression of RIPK3 in primary renal tubular epithelial cells was detected using immunofluorescence staining. DAPI is nuclear staining; red fluorescence indicates RIPK3 fluorescent protein; Merge is an overlapping combination of the two (Scale bar = 10 μm).

RIPK3 is a key component of the necrotic apoptotic vesicles [24,25] and the primary purpose of RIPK3 signaling is to activate MLKL and initiate necroptosis [26,27]. Immunofluorescence was used to determine whether wogonin had a regulatory effect on the release of RIPK3 induced by NIBV. The results indicate that compared to the uninfected group, the red fluorescence intensity of the infected group cells was significantly increased, and after the intervention of wogonin, the red fluorescence intensity of cells decreased significantly (Figure 5). Therefore, the immunofluorescence of RIPK3 in the NIBV-treated renal tubular epithelial cells further confirmed the inhibitory effect of wogonin on RIPK3.

### 2.7. Effect of Wogonin on Mitochondrial Membrane Permeability

**Figure 6:**
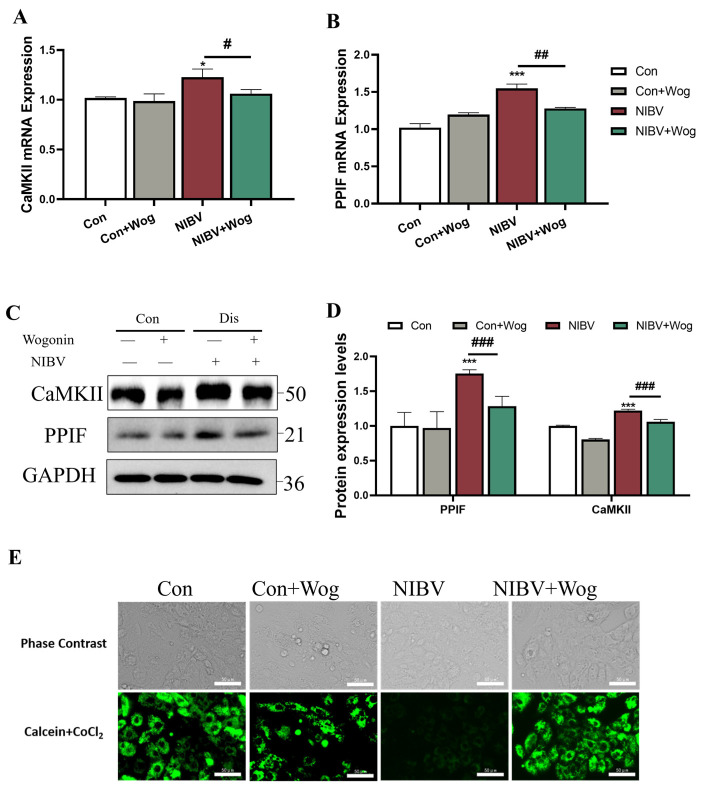
Effect of wogonin on mitochondrial membrane permeability. (**A**,**B**) The mRNA levels of CaMKII and PPIF in renal tubular epithelial cells (*n* = 3). (**C**) CaMKII and PPIF protein levels in renal tubular epithelial cells after NIBV infection. Results are quantified and analyzed in (**D**) (*n* = 3). (**E**) Effects of wogonin on mitochondrial membrane potential decline after viral infection (Scale bar = 50 μm). “*” indicates the comparison with the Con group (* *p* < 0.05, *** *p* < 0.001), “#” indicates the comparison with the NIBV group (# *p* < 0.05, ## *p* < 0.01, ### *p* < 0.001).

Under normal physiological conditions, MPTP (mitochondrial permeability transition pore) is in a short-term open state. If it remains continuously open, it can cause MPT (mitochondrial permeability transition), leading to a disorder in mitochondrial membrane potential. Ca^2+^ overload is an important factor in promoting the sustained opening of MPTP. In our study, NIBV infection increased the mRNA expression levels of CaMKII (Calmodulin dependent protein kinase II) and PPIF (cyclophilin D, Cyp-D) (*p* < 0.05 or *p* < 0.001), and wogonin could alleviate the increase in expression caused by NIBV infection (*p* < 0.05 or *p* < 0.01), and its protein expression was consistent with mRNA results (Figure 6A–D). Meanwhile, the MPTP kit was used to detect the changes in the opening of the mitochondrial permeability transition pore. The mitochondrial inner membrane maintains a normal membrane potential gradient, which is crucial for cellular respiration and energy supply. However, as cells undergo apoptosis or death, the opening of the mitochondrial permeability transition pore triggers marked alterations in mitochondrial permeability. Under normal circumstances, the MPTP of mitochondria is closed, and CoCl_2_ cannot enter mitochondria. Therefore, after Calcein AM staining, CoCl_2_ treatment will lead to only Calcein green fluorescence in mitochondria. The degree of green fluorescence indicates the level of openness, with stronger fluorescence indicating lower openness and weaker fluorescence indicating higher openness. In the Con group, the cytoplasm including mitochondria emitted strong green fluorescence. Treatment of cells with NIBV induced excessive Ca^2+^ into the mitochondrial matrix, leading to the opening of MPTP and quenching of Calcein green fluorescence. After the application of wogonin, the green fluorescence intensity observed in Figure 6E was notably higher compared to the NIBV group. This indicated that wogonin can alleviate the abnormal opening of mitochondrial membrane permeability pores caused by NIBV.

## 3. Discussion

In recent years, NIBV has had a significant impact on the poultry industry, and currently there is no effective specific therapeutic drug available. As a result, the development of effective anti-NIBV drugs holds paramount importance. Viral infections can cause cell death, but the mechanism of NIBV-induced cell death and regulatory drugs have not yet been found. Wogonin is a flavonoid compound with a good antiviral effect. This study aimed to explore how wogonin protects against apoptosis and necrosis in renal tubular epithelial cells triggered by NIBV infection. These results indicated that NIBV-induced cell death and wogonin had a significant inhibitory effect. Wogonin alleviated NIBV-induced cell death (apoptosis and necroptosis), elevated the decline in mitochondrial membrane potential, decreased the opening of mitochondrial permeability transition pores, and downregulated the expression levels of apoptosis and necrosis-related factor mRNAs and proteins. This study could provide valuable insights for the development of drugs against NIBV. Wogonin is a potential drug candidate for inhibiting NIBV by suppressing apoptosis and necroptosis.

Multiple studies have shown that flavonoid drugs have antiviral effects [28,29]. Wogonin, a flavonoid compound, has been found to possess antiviral activity. Flavonoid phytochemicals can inhibit viruses and act on them through various mechanisms, for example by blocking virus attachment, entry into cells, hindering different stages of protein translation, viral DNA replication and polyprotein processing [30]. In this study, we found that wogonin inhibited viral replication in renal tubular epithelial cells, resulting in reduced cell death.

Apoptosis is a crucial mechanism for inhibiting viral replication and clearing virus-infected cells [31]. Studies have shown that porcine circovirus type 2 (PCV2) [32], swine enteric coronavirus (SeCoV) [14] and other viruses can induce apoptosis. Apoptosis is characterized by exogenous and endogenous pathways [33]. The exogenous pathway is mediated through the activation of death receptor family members [34], while the endogenous pathway is mainly regulated by the release of mitochondrial CYT-C [35]. Cytochrome c, upon release from the mitochondria, binds with Apaf-1 (apoptotic protease activating factor-1) and the precursor of Caspase-9 to form an apoptosome, thereby activating Caspase-9 [36]. Upon activation, Caspase-9 initiates the cleavage and activation of pro-Caspase 3/7, ultimately resulting in cell death through the degradation of various endogenous substrates [37]. This process is accompanied by changes in mitochondrial membrane potential [38]. The results of this experiment confirm that NIBV is capable of inducing apoptosis in chicken renal tubular epithelial cells, and that wogonin can inhibit this process. Flow cytometry results demonstrated that apoptosis occurred in renal tubular epithelial cells following infection, and that wogonin had an inhibitory effect. To confirm our results, we analyzed the changes in mitochondrial membrane potential of renal tubular epithelial cells after NIBV infection and wogonin intervention, as well as the levels of mRNA and protein apoptosis factors such as CYT-C, Caspase-3, Caspase-9, etc. The results showed a significant reduction in the mitochondrial membrane potential of renal tubular epithelial cells, as well as a significant increase in the mRNA and protein expression levels of apoptosis-related factors. Wogonin was found to have a significant improvement effect. These discoveries have bridged the knowledge gap in investigating the apoptosis of chicken renal tubular epithelial cells and elucidating the function of wogonin in NIBV infection. According to reports, wogonin possesses anti-inflammatory properties, which can protect cells from damage and death, including necrosis and apoptosis [39].

Many viral infections, including Influenza A virus (IAV) [12] and human herpes simplex virus (HSV) [40], can induce necroptosis. Studies have shown that flavonoids can reduce necroptosis. By suppressing oxidative stress and the MAPK/NF-κB pathway, rutin effectively mitigates cadmium-induced necroptosis [41]. Hesperidin improves intestinal epithelial barrier damage by inhibiting RIPK3/MLKL-mediated necroptosis [42]. Quercetin effectively inhibits the shift of macrophages/microglia toward the M1 phenotype polarization by inhibiting the STAT1 and NF-κB pathways, resulting in reduced necroptosis of OLs [43]. Several studies have demonstrated that wogonin also has an effect on programmed cell death. It can reverse damage by inhibiting programmed cell death, and can also promote tumor cell death by promoting programmed cell death to achieve cancer treatment [44,45]. However, whether the flavonoid compound wogonin can play a role in NIBV-induced necroptosis has not been reported. In this study, we detected the mRNA (TNF-α, TRADD, FADD, MLKL, RIPK1, RIPK3) and protein (p-RIPK3/RIPK3, p-MLKL/MLKL, RIPK1) expression of necroptosis-related factors by flow cytometry. This study found that NIBV induces necroptosis of renal tubular epithelial cells, and obvious inhibitory effects were observed after intervention with wogonin. RIPK3 is identified as a key molecule in necroptosis and a crucial target for the regulation of this condition and its associated diseases [46]. Studies have shown that flavonoids have an effect on RIPK3. Compounds that inhibit RIPK3 can prevent TNF-induced necroptosis at high concentrations [47]. However, it has not been reported whether the flavonoid wogonin can exert antiviral effects by inhibiting RIPK3. We found that wogonin has an anti-NIBV effect through the expression of necroptosis-related genes and proteins, so we hypothesized that wogonin can alleviate NIBV-induced necroptosis by regulating RIPK3. The immunofluorescence experiment of RIPK3 showed that wogonin significantly inhibited the increase of RIPK3 expression caused by NIBV infection. Previous studies have also found that wogonin can alleviate myocardial cell apoptosis and necrosis caused by diabetes [48]. In line with our findings, wogonin decreased necroptosis in renal tubular epithelial cells caused by NIBV infection.

Studies have shown that CaMKII is a substrate of RIPK3, which can promote the opening of mitochondrial membrane permeability transition pores, thereby mediating necrotic apoptosis of cardiomyocytes [49]. Ca^2+^ also regulates programmed cell death mechanisms, including apoptosis [50]. Furthermore, intrinsic stimuli such as calcium overload and DNA damage can cause the cell-dependent apoptosis pathway. Ca^2+^ overload can cause the opening of mPTP, leading to the release of CYT-C triggering mitochondrial apoptosis [51]. To investigate this, our study employed a combination of mRNA and protein CaMKII expression level measurement and mPTP staining assessment. Our study found that NIBV infection resulted in an abnormal elevation of calmodulin CaMKII and an abnormal widening of the mitochondrial membrane permeability transition pore. Wogonin was found to significantly reverse this phenomenon. Based on these findings, there is speculation that the association between necroptosis and mitochondrial apoptosis may be due to the abnormal opening of the mitochondrial membrane permeability transition pore caused by calcium overload resulting from necroptosis. Additional research is required to verify this hypothesis.

## 4. Materials and Methods

### 4.1. Cell Culture

Chicken kidneys were harvested and digested in a 37 °C water bath using Type I collagenase purchased from Solarbio for a few minutes. Following digestion, the process was terminated with a serum-containing medium, and the tissue was gently dispersed using a pipette gun to separate the cells. The filtrate was then collected by filtering it through a cell sieve (Solarbio, Beijing, China). The supernatant was discarded by centrifugation and centrifuged multiple times with PBS (Solarbio, Beijing, China) to achieve a cleaning effect. The collected cells were suspended in DMEM containing 15% FBS (ExCell, South America, Uruguay) and 1% Penicillin-Streptomycin Liquid (Solarbio, Beijing, China). Subsequently, the cells were seeded and incubated in a 37 °C incubator.

### 4.2. Virus and Drug

This study used the NIBV (SX9) strain preserved by the College of Animal Science and Technology, Jiangxi Agricultural University. Wogonin (HY-N0400, C_16_H_12_O_5_, 99.98% purity) was obtained from MedChemExpress (MCE, Monmouth Junction, NJ, USA).

### 4.3. Antibodies and Reagents

The following antibodies were used for western blot: Anti-P-MLKL (T56238, Abmart, Baltimore, MD, USA), MLKL (sc-293201, Santa, Dallas, TX, USA), Anti-P-RIPK3 (T57436, Abmart, Maryland, USA), Anti-RIPK3 (sc-374639, Santa Dallas, TX, USA), Anti-RIPK1 (WL04522, Wanleibio, Beijing, China), Anti-CYT-C (WL02410, Wanleibio, Beijing, China), Anti-Caspase-3 (WL03117, Wanleibio, Beijing, China), Anti-Caspase-9 (WL03421, Wanleibio, Beijing, China), Anti-CaMKII (WL03453, Wanleibio, Beijing, China), Anti-PPIF (TD3147, Abmart, Baltimore, MD, USA), GAPDH (60004-1-lg, Proteintech, Rosemont, IL, USA), Rabbit anti-Goat IgG serum (B900710, Proteintech, Rosemont, IL, USA), and Mouse anti-Goat IgG serum (B900730, Proteintech, Rosemont, IL, USA).

### 4.4. The Amount of Virus Replication

To extract total RNA from chicken renal tubular epithelial cells using the Trizol method and perform reverse transcription using the high-quality RNA reverse transcription kit purchased from TransGen Biotech. Then, the cDNA was saved at −20 °C for use. The viral load copy number of the extracted cells was quantitatively detected using fluorescence quantitative PCR.

### 4.5. Quantitative Real-Time Polymerase Chain Reaction Analysis

Following the manufacturer’s guidelines provided by TransGen Biotech, located in Beijing, China, total RNA was extracted from chicken kidney tubular epithelial cells, and reverse transcription was performed. The resulting cDNA was saved at −20 °C for follow-up experiments. Primers specifically amplifying segments of CYT-C, APAF1, Caspase-3, Caspase-9, TNF-α, TRADD, FADD, RIPK1, RIPK3, MLKL, CaMKII, APAF1 and PPIF were designed using Primer 8.0. The primer sequences are provided in Table 1. GAPDH was used as the housekeeping gene, and the 2^−ΔΔ^Ct method was employed for data analysis. Experimental results were visualized using GraphPad Prism 8.0.

### 4.6. Western Blotting Analysis

Cell samples were collected with a solution containing RIPA lysate, phosphatase inhibitor, and protease inhibitor. Using the Solarbio BCA Protein Assay Kit, the total protein concentration was determined. First, the samples underwent processing via SDS-PAGE and then were transferred to a PVDF membrane sourced from EMD Millipore in Billerica, MA, USA. Then, the membrane was sealed using a quick blocking solution and washed with PBST. Subsequently, the membrane was incubated with the primary antibody for roughly 18 h, maintaining a temperature of 4 °C. After washing away the excess primary antibody with PBST, the membrane was further incubated with the corresponding secondary antibody for a duration of 45 min. Finally, imaging analysis was performed using a protein imaging system.

### 4.7. Cell Viability Assay

Renal tubular epithelial cells were seeded into cell culture clusters for 24 h and subsequently treated with varying concentrations (0, 0.625, 1.25, 2.5, 5, and 7.5 μM/mL) of wogonin (MCE, New Jersey, USA) for 24 h (*n* = 12). Next, CCK-8 (Yeasen Biotechnology, Shanghai, China) was added at 10% volume to the medium and it was then incubated for 3 h at 37 °C. The absorbance was determined using a microplate reader (PerkinElmer, Waltham, MA, USA).

### 4.8. Flow Cytometry Analysis

After collecting the cultured cells, they were processed using the Annexin V-Fluorescein Isothiocyanate (FITC)/Propidium Iodide (PI) staining kit (Beyotime Biotechnology, Shanghai, China) to assess cell status and death. Subsequently, a flow cytometer was utilized for the detection of these indicators. During this detection process, please take care to avoid exposure to light.

### 4.9. Immunofluorescence Microscopy

After discarding the culture medium from the cultured renal tubular epithelial cells, they were fixed with 4% paraformaldehyde for 15 min. Initially, the cells underwent a blocking process utilizing an immunostaining blocking solution for a duration of 60 min. Next, the primary antibody RIPK3 was added to incubate the cells and combined with the secondary antibody. Then, they were counterstained with DAPI for 3 min and washed with PBS. Finally, the anti-quenching agent was added dropwise and photographed with a fluorescence microscope (OLYMPUS, CKX41, Tokyo, Japan).

### 4.10. Mitochondrial Membrane Potential (JC-1)

Discard the culture medium and collect the cells in a tube. 500 μL of JC-1 staining working solution from Beyotime Biotechnology in Shanghai, China was added to each tube, thoroughly mixed, and then incubated at 37 °C for a period of 20 min. Subsequently, an appropriate amount of JC-1 staining buffer (1×) was added for washing. Finally, cell culture medium was added and the samples were placed on glass slides for observation under a fluorescence microscope (OLYMPUS, CKX41, Japan).

### 4.11. MPTP Assay

To assess the extent of mitochondrial permeability transition pore opening, a mitochondrial permeability transition pore assay kit purchased from Beyotime Biotechnology was used. After removing the culture medium, the cells were washed 1–2 times with PBS. Staining solution and fluorescence quenching working solution were added, and the cells were incubated in a dark place at 37 °C for 35 min. After incubation, preheated cell culture medium was added and the cells were incubated at 37 °C in the dark for 30 min. After removing the medium, the cells were washed three times with PBS. Next, detection buffer was added and the samples were observed under a fluorescence microscope (OLYMPUS, CKX41, Japan), while avoiding light throughout the process.

### 4.12. Statistical Analysis

The experimental data are in accordance with a normal distribution and were analyzed by SPSS 26.0 for Bonferroni’s and Tukey’s tests. The final statistical results are expressed as mean ± standard deviation (SD) and visualized using GraphPad Prism 8.0. The symbol “*” indicates statistically significant differences compared with the Con group (* *p* < 0.05, ** *p* < 0.01, *** *p* < 0.001). The symbol “#” indicates significant differences between the NIBV group and the NIBV+Wog group (^#^ *p* < 0.05, ^##^ *p* < 0.01 and ^###^ *p* < 0.001).

## 5. Conclusions

In this study, we have shown that NIBV infection can trigger apoptosis and necroptosis, and wogonin can reduce cell death by inhibiting apoptosis and necroptosis. Collectively, it provides a theoretical basis for the prevention and treatment of renal injury caused by NIBV, and serves as a guide for developing drugs aimed at preventing and treating NIBV infections.

## Figures and Tables

**Table 1:** The sequences of primers for the target genes.

Gene	Forward 5′–3′	Reverse 5′–3′
CYT-C	TCCCAGTGCCATACGGTTAA	TGTTTTCGTCCAAACAGGC
APAF-1	AAACAGGACAAAGCAGGGT	CGTATTTGCGCACATCAGCA
Caspase-3	AAAGATGGACCACGTCAGG	GTCCGGTATCTCGGTGAAGT
Caspase-9	TCGTGGTCATCCTCCCCAT	TTCCTCTCAAACTCGGGCAC
PPIF	AGAGCGCTTTGTATGGTGAA	TGGCCATTGAAGAACACCA
CaMKII	CCACCACAATGGGGTTGTC	TAGGGATCCTTCGGAGGAC
TNF-ɑ	ATACTGTGTGTGGCGTCGG	AAGCACTCTTCTCCAACGCA
TRADD	ATGAATGGTGTGGACGTCT	TGGGGGTTCAGTGACATGC
FADD	GAATTCTTTGTCCGCCACC	CTCTCCAACGTTCTCGCAT
RIPK1	CAGCTGCCTTCTGTTCCTAC	TGCTGAAGCCTAGTTCACGG
RIPK3	ACTTGCAGCCTGCAATTCAG	TTGCTGTGAGCGAAGGATGT
MLKL	CATTTGAAGGCTGCCTCTCC	GAAGGCCCGACACTGATTGA
GAPDH	TGGCATCCAAGGAGTAGC	GGGGAGACAGAAGGAACAG

## Data Availability

No new data were created or analyzed in this study.
